# Prognostic Significance of CD163+ and/or CD206+ Tumor-Associated Macrophages Is Linked to Their Spatial Distribution and Tumor-Infiltrating Lymphocytes in Breast Cancer

**DOI:** 10.3390/cancers16112147

**Published:** 2024-06-05

**Authors:** Canbin Fang, Maisy Y. Cheung, Ronald C. Chan, Ivan K. Poon, Conrad Lee, Curtis C. To, Julia Y. Tsang, Joshua Li, Gary M. Tse

**Affiliations:** 1 State Key Laboratory of Translational Oncology, Department of Anatomical and Cellular Pathology, Prince of Wales Hospital, The Chinese University of Hong Kong, Hong Kong SAR, China; 2 Department of Pathology, The University of Hong Kong, Hong Kong SAR, China

**Keywords:** breast cancers, CD163, CD206, tumor-associated macrophage

## Abstract

**Simple Summary:**

The manual assessment and identification of tumor-associated macrophages (TAMs) with a single marker limits a thorough analysis of their spatial distribution and density. We applied a digital workflow to compare the features of TAM populations identified by CD163 and CD206 and showed that these markers highlighted TAM populations with distinct clinical implications. The spatial distribution of CD163 TAMs and their interactions with tumor-infiltrating lymphocytes (TILs) refined the prognostic value in breast cancer. Conversely, CD206 TAMs may not have any unfavorable prognostic impact.

**Abstract:**

Tumor-associated macrophages (TAMs) is a key element in the breast tumor microenvironment. CD163 and CD206 have been utilized for TAM identification, but the clinical implications of TAMs identified by these markers have not been thoroughly explored. This study conducted a comparative analysis of CD163 and CD206 TAMs using digital image analysis, focusing on their spatial distribution and prognostic significance in relation to tumor-infiltrating lymphocytes (TILs). Distinct clinico-pathological and prognostic characteristics were noted between the two types of TAMs. CD163 TAMs were linked to high-grade tumors (*p* = 0.006), whereas CD206 TAMs were associated with a higher incidence of nodal metastasis (*p* = 0.033). CD206 TAMs were predominantly found in the stroma, with more cases being stromal CD206-high (sCD206-high) than tumoral CD206-high (tCD206-high) (*p* = 0.024). Regarding prognostication, patients stratified according to stromal and tumoral densities of CD163 showed different disease-free survival (DFS) time. Specifically, those that were sCD163-low but tCD163-high exhibited the poorest DFS (chi-square = 10.853, *p* = 0.013). Furthermore, a high sCD163-to-stromal-TILs ratio was identified as an independent predictor of unfavorable survival outcomes (DFS: HR = 3.477, *p* = 0.018). The spatial distribution and interactions with TILs enhanced the prognostic value of CD163 TAMs, while CD206 TAMs appeared to have limited prognostic utility in breast cancer cases.

## 1. Introduction

Previously, the significance of the tumor microenvironment (TME) in breast cancer development and treatment response has gained increasing recognition [1]. Tumor-associated macrophages (TAMs) are a key component of the TME, constituting over 50% of tumor mass in some cases [2]. Unlike the proinflammatory M1 polarized macrophages [3], TAMs demonstrate properties more akin to M2 polarized macrophages, known for their immunosuppressive functions, support of wound healing, and contribution to tumor progression and therapy resistance [4]. In various cancers, including breast cancer, the presence of TAMs has been linked to unfavorable outcome [5].

In earlier studies, TAMs were identified by CD68, a pan-macrophage marker expressed by both M1 and M2 polarized macrophages [6]. Subsequently, CD163, a scavenger receptor, and CD206, a C-type mannose receptor 1, upregulated in M2 macrophages have been used as TAM markers [7,8,9,10,11]. Despite their common expression in M2 macrophages, these markers may represent different subsets of TAMs. The CD163 level is increased in response to IL-10, while CD206 is upregulated by IL-4 and IL-13 [12]. Therefore, the TAM subsets represented by the two markers could be associated with a specific cancer milieu and found in cancers with different features. Notably, there were differential clinico-pathological associations of CD163 and CD206 TAMs. CD163 TAMs were positively associated with a higher tumor grade, while CD206 TAMs were associated with smaller tumor size in triple-negative breast cancer (TNBC) [13]. Moreover, a high density of CD163 was associated with poor outcomes in breast cancer in several studies [14]. However, the outcome association of CD206 TAMs is less well-studied and showed inconsistent results [13,15]. Most TAM studies to date used a single marker, with few examining multiple markers on tissue microarrays [16]. Studies comparing CD163 and CD206 TAMs in breast cancer are lacking. The most clinically relevant TAM subset in breast cancer has not been fully examined.

More recently, a better prognostication was suggested with the combined assessment of TAMs and tumor-infiltrating lymphocytes (TILs) in breast cancers. TNBC with high TAMs (CD68/CD163) and low TILs, but not those with high TILs, was associated with poor patient survival [17]. HER2+ breast cancers with a high CD8-TIL-to-CD68-TAM ratio derived more benefit from anti-HER2 treatment [18]. Additionally, the spatial localization of TAMs could also affect the prognosis. The distance of CD163 TAM from cancer cells and a higher average number of them in close contact with cancer cells were independent predictors of unfavorable prognosis [19]. Interestingly, in gastric cancers, the spatial locations of CD163 and CD206 TAMs were related to their proximity to tumor cells and associated with specific environmental gene signatures and PD-L1 expression [20], implying an interactive process between cancer cells and the TME. For the CD163 and CD206 TAMs, their spatial significance and combined assessment with TILs remain to be explored.

This study compared the clinical significance of CD206 and CD163 TAMs in a series of well-characterized breast cancers. Further prognostic assessment was conducted on their spatial distribution and interactions with TILs. Manual counting and a semi-quantitative analysis have been used for TAM evaluation in most previous studies [13,15,21]. The analysis is labor-intensive and prone to inter-/intra-rater variations. Here, a digital analysis approach was adopted for a more detailed and objective quantitative assessment on whole-tissue sections.

## 2. Materials and Methods

### 2.1. Patients

Invasive breast carcinoma patients from 2005 to 2008 at Prince of Wales Hospital, the Chinese University of Hong Kong (PWH, CUHK), were included in this retrospective study. All the excision specimens were formalin-fixed, paraffin-embedded (FFPE) with routine tissue processing. The hematoxylin and eosin (H&E)-stained slides were reviewed to confirm diagnosis (WHO classification) [22] and grade (modified Bloom and Richardson) [23]. Patient particulars and clinical data were retrieved from medical records, including the patient’s age, tumor size, lymph node involvement, pT and pN stages, and patient’s outcome data. In addition, stromal TILs (sTILs), fibrotic focus, necrosis, apocrine and lymphovascular invasion (LVI) were evaluated as previously reported [24]. Regarding the outcome data, disease-free survival (DFS) was defined as the duration from the date of initial diagnosis to the first detection of breast cancer-specific relapse or death. Overall survival (OS) was defined as the time interval from the date of initial diagnosis to the date of death. The study was approved by the Joint Chinese University of Hong Kong New Territories East Cluster Clinical Research Ethics Committee. The effect on survival was assessed based on REMARK criteria [25].

### 2.2. Immunohistochemistry (IHC) Staining of Tumor Sections

IHC on four-micron freshly cut FFPE sections was carried out using the Ventana BenchMark ULTRA system (Ventana, Tucson, AZ, USA) after deparaffinization, rehydration, and antigen retrieval. Antigen retrieval was carried out using Cell Conditioning Solution (CC1, Tris-based EDTA buffer, pH 8.0; Ventana, Tucson, AZ, USA). The sections were stained with antibodies against CD163 (clone 10D6) and CD206 (clone 5C11). The signals were detected using the Ultraview Universal DAB Detection Kit (Ventana, Tucson, AZ, USA). Sections were counterstained by hematoxylin, dehydrated and mounted manually. The slides were scanned by a Leica Aperio GT 450 scanner (Leica Biosystems, Danvers, MA, USA) at 400× magnification. The whole slide images were analyzed using our established digital pipeline.

For all other markers, including ER, PR, HER2, Ki67, EGFR, c-Kit, P63, CK5/6, CK14, and PDL1, the results were retrieved from our database based on our previous tumor microarray analysis. Details on antibody clones, staining conditions, and assessments are shown in Supplementary Table S1. IHC surrogates for molecular subtype classification are as follows: luminal A (ER+, PR ≥ 20%, HER2−, Ki67 < 20%), luminal B (ER+, PR < 20%/and HER2+/and Ki67 ≥ 20%), HER2 over-expressed (HER2-OE; ER−, PR−, HER2+), and TNBC (ER−, PR−, HER2−).

### 2.3. Digital Image Analysis by QuPath

Digital microscopic images were imported into QuPath for tumor recognition and cell detection analysis (Supplementary Figure S1). Five regions of interest (ROI) of 1.96 mm^2^, including a hotspot stromal region, a hotspot tumor region, and three representative stromal–tumor interfaces, were selected. Hotspot areas were identified by examining the entire section under low-power magnification for stromal and tumor regions with the highest staining. The same ROIs were applied to CD206 and CD163 IHC stained images from the same case. The tumor and stromal regions were annotated using a model-assisted approach. Tumor and stromal areas were identified by a cancer detection model for breast cancers, which was developed based on our previous studies [26]. By adjusting the thresholds of probability, at least nine predicted tumor labels were generated for each sample. The best-fit tumor mask for each case was selected manually. The analysis of the stromal area was limited to 50 μm from the tumor to avoid missing areas in some ROIs. For the detection of CD163 and CD206 TAMs, a similar digital workflow as described previously by QuPath [24], which comprised color deconvolution, cell detection and segmentation, and immunostaining expression detection, was adopted. CD163 and CD206 TAMs were automatically detected based on the optical density of DAB staining. The accuracy of the digital workflow for tumor detection and IHC assessment was verified by a pathologist. The parameters used for cell detection and IHC assessment are listed in Supplementary Table S2. The total cell counts and coordinates of CD206/CD163 TAMs and areas in tumor and stroma regions for each ROI were exported. The average density of CD163 and CD206 TAMs from all ROIs in different regions, i.e., CD163 and CD206 densities in the tumor nest (tCD163 and tCD206) and the stroma (sCD163 and sCD206) were calculated accordingly. The average distance of sCD163/CD206 TAMs from the tumor nest was also evaluated. Median values were adopted as cutoffs to categorize the cohort into TAM-high and -low groups.

### 2.4. Statistical Analysis

The data were analyzed using the statistical software SPSS for Windows, Version 27. Spearman’s rank-order correlation was utilized to correlate tumoral and stromal CD163 and CD206 and sTIL as continuous variables. Wilcoxon signed-rank test was used to compare the density of CD163 and CD206 in the tumor and stroma. CD163 and CD206 expressions were compared for the clinic-pathological features, biomarkers expressions, and molecular subtypes as categorical variables by chi-square analysis or Fisher’s exact test. The Mann–Whitney U test was applied for analysis of the differences in patient’s age and tumor size with CD163 and CD206 expressions. Survival data were evaluated by multivariate cox regression analysis and the Kaplan–Meier method. Statistical significance was established at *p* < 0.05.

## 3. Results

### 3.1. Cohort Features

A total of 225 invasive breast cancer cases were included in this cohort, including 16 cases of grade I, 124 cases of grade II and 85 cases of grade III cancers, with a median patient age of 47 (mean 48.6, range 29–82) and a median tumor size of 1.8 cm (mean 2.09, range 0.1–7.6 cm). Among them, there were 219 invasive breast carcinomas of no special type (IBC-NST), 4 invasive lobular carcinomas (ILC), 1 mixed ILC/IDC, and 1 mucinous carcinoma. Based on the results of IHC, 52 cases were classified as luminal A, 123 cases as luminal B, 21 cases as HER2-enriched, and 26 cases as TNBC. Three cases were not classified due to incomplete IHC data.

### 3.2. Correlations of CD163 and CD206 TAM Density with Clinico-Pathological and Biomarker Features in Different Regions

**Figure 1:**
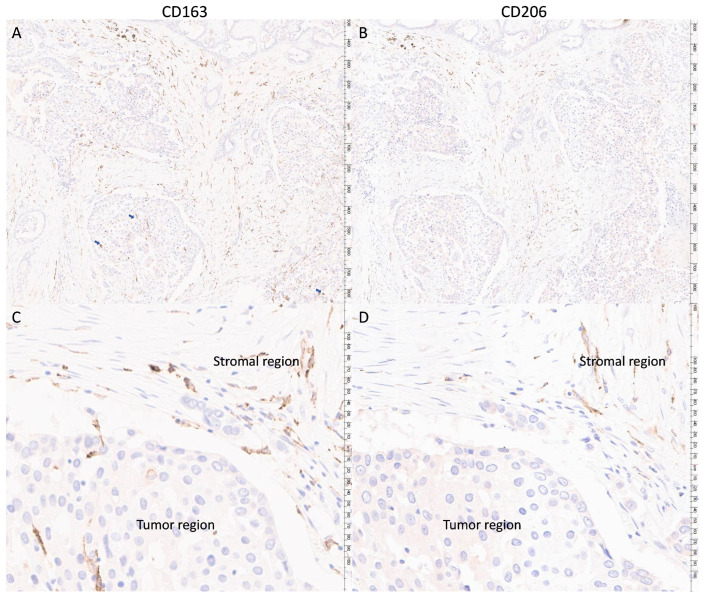
Representative staining (brown color) of CD163 and the corresponding staining of CD206 from the same patient. Panels (**A**,**B**) showed 40× magnification (major unit at scale: 100 microns) and panels (**C**,**D**) showed 200× magnification (major unit at scale: 10 microns). The presence of CD163 TAM appears to be more notable in tumoral region than CD206 TAM (blue arrows indicated the CD163 TAM in tumoral regions).

CD163 and CD206 expression was successfully assessed in 220 and 211 cases, respectively (Figure 1 and Supplementary Figure S2). Overall, the median stromal density for CD163 (sCD163) and CD206 (sCD206) TAMs were 354.1 cells/mm^2^ (mean 538.7, range 2.3–3398.0 cells/mm^2^) and 303.2 cells/mm^2^ (mean 409.2, range 9.3–2707.8 cells/mm^2^), respectively. The median tumoral density for CD163 (tCD163) and CD206 (tCD206) TAM were 210.8 cells/mm^2^ (mean 373.4, range 1.2–4037.4 cells/mm^2^) and 111.6 cells/mm^2^ (mean 177.0, range 2.9–2221.6 cells/mm^2^), respectively (Supplementary Figure S3). Cases were categorized into high- and low-TAM subgroups based on the median density value. sCD163 and tCD163 TAMs showed a stronger association with each other than CD206 TAMs (rs = 0.718 vs. 0.534). CD163 and CD206 TAMs only correlated with each other moderately in the stroma or tumor nest (rs = 0.493 and 0.483, respectively). When categorized as high and low subgroups, there were more sCD206-high, tCD206-low cases (*p* = 0.024), but not for CD163. A trend of more tCD163-high than tCD206-high was found (*p* = 0.066) (Supplementary Table S3 and Supplementary Figure S3).

In line with their correlations, the clinico-pathological associations of the CD163 and CD206 TAM density shared some similarities. Regardless of their locations, both TAMs showed significant associations with a larger tumor size and high TIL level (*p* ≤ 0.016). The stromal density of both were associated with the presence of necrosis, LVI, high Ki67, HER2 positivity, and PDL1+ immune cells (*p* ≤ 0.032). However, tCD163 showed associations with a higher grade, high Ki67, HER2 positivity, and PDL1+ immune cells (*p* ≤ 0.038), but not for tCD206. Additionally, sCD163 was associated with a higher grade, ER negativity, PR negativity, P63 positivity and a differential distribution in molecular subtypes (*p* ≤ 0.012), but not for sCD206. The sCD163 distribution was found to be the highest in HER2-OE (80.9%), followed by TNBC (64.0%), luminal B (50.4%) and luminal A (30.6%). Only sCD206 showed a positive association with nodal metastasis (*p* = 0.033). The tCD206 showed associations with ER negativity, PR negativity, and CK14 (*p* ≤ 0.022) positivity, but not sCD206 (Table 1 and Supplementary Figure S4).

### 3.3. Correlations of sCD163 and sCD206 TAM Distance from Tumor Nest with Clinico-Pathological and Biomarker Features

For a more detailed spatial analysis, we next examined the proximity of the different TAMs from the tumor nest. The median distances between sCD163 and sCD206 TAMs and the tumor nests were 22.99 μm (mean = 22.84, range 4.80–30.92 μm) and 24.07 μm (mean = 23.95, range 16.21–29.63 μm), respectively (Supplementary Figure S5). sCD206 TAMs were located significantly closer to the tumor than sCD163 TAMs, particularly in the luminal B HER2-OE and TNBC subtypes (Supplementary Figure S6, *p* ≤ 0.024). Their distances from the tumor nest correlated positively with each other (rs = 0.642, *p* < 0.001). For the clinico-pathologic association, the sCD163-tumor distance showed negative associations with grade, sCD163, tCD163, and tCD206 densities (*p* ≤ 0.048), while the sCD206-tumor distance was associated negatively with ER, PR, and tCD206 densities (*p* ≤ 0.045) (Table 2, Supplementary Figure S4). No associations were found with the other features.

### 3.4. Survival Analysis

**Figure 2:**
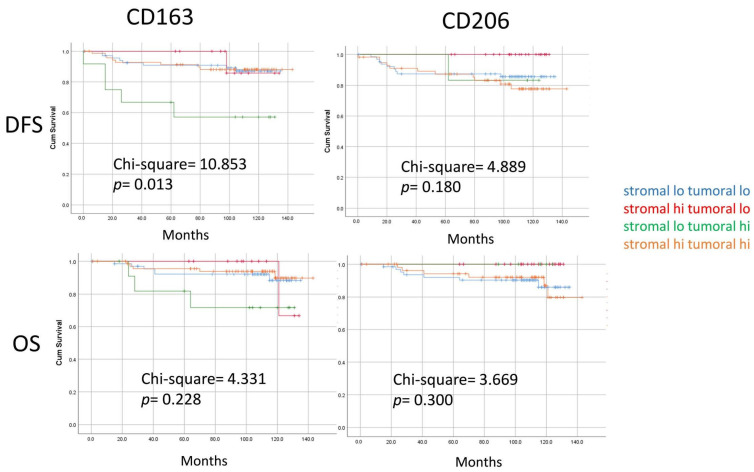
Kaplan–Meier analysis according to grouping based on TAM density in both stromal and tumor compartments. Patients were stratified based on the density of two TAMs in different compartments. Patients with stromal-low, tumoral-high CD163 TAM showed significantly worse DFS. Difference in survival curves were assessed with a log-rank test.

**Figure 3:**
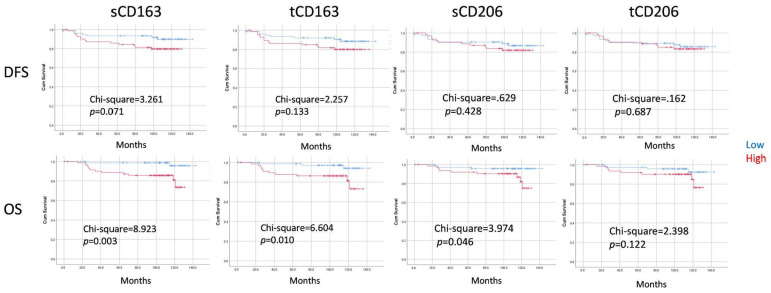
Kaplan–Meier analysis of DFS and OS according to the TAM density in relation to sTIL. Patients were stratified into two subgroup based on the median TAM density/sTIL ratio. High stromal TAM/sTIL showed significantly worse. Difference in survival curves were assessed with a log-rank test.

Follow-up data were available for 163 patients, with a median follow-up time of 110 months (ranging from 1–143 months). Among them, there were 23 death/relapses (14.1%). Kaplan–Meier analyses revealed that the densities of sCD206, sCD163, tCD206, and tCD163 TAMs or the distance of sCD163/sCD206 to the tumor nest on their own showed no correlations with prognosis in the overall cohort or ER− and ER+ subgroups (Supplementary Figures S7 and S8). Patients were further classified into four groups according to TAM density in stromal and tumoral compartments. Differences in DFS were found for CD163 TAM subgroups (chi-square = 10.853, *p* = 0.013). Particularly, the small subset of patients who had low sCD163 but high tCD163 had the worst DFS. A similar trend was also noted in OS (Figure 2 and Supplementary Figure S9). Next, we also explored the prognostic value of relative proportion of different TAMs and TILs. The ratios of TAM density to sTIL were evaluated, and the median score was used as a cutoff. High relative scores of sCD163, tCD163 and sCD206 to sTIL were significantly associated with worse OS (chi-square = 8.923, *p* = 0.003; chi-square = 6.604, *p* = 0.010; and chi-square = 3.974, *p* = 0.046, respectively) (Figure 3). Significant differences for CD163 TAM/sTIL were also observed in both ER+ and ER- subsets, particularly for OS (Supplementary Figure S10). Multivariate cox regression analysis revealed that the relative score of sCD163 to sTIL was a poor independent prognostic feature for OS (HR = 3.477, *p* = 0.018). A trend was observed in DFS (HR = 1.671, *p* = 0.058) (Table 3).

## 4. Discussion

TAMs represent a diverse group of immune cells that infiltrate the tumor microenvironment. Different markers, including CD206 and CD163, were used to identify TAMs, and only very few studies have compared the clinical values of these markers. In this study, we compared the features of CD163 and CD206 TAMs in breast cancers. While both TAMs shared some similarities in their clinico-pathological associations, such as the association with larger tumor size and high levels of TILs, differences were also observed. Only CD163 TAMs were associated with a higher grade, different molecular subtypes, and hormonal receptor negativity. For CD206 TAMs, their stromal density showed an association with nodal metastasis. The two TAMs also appeared to be located differently. CD163 TAMs showed a strong correlation between their stromal and tumoral locations and infiltrated into the tumor nest. In contrast, CD206 TAMs tend to be located in the stroma. The lack of CD206 TAMs present within the tumor body in breast cancers was also reported previously [27]. A similar preferential location of these two TAMs has been demonstrated in gastric cancers [20]. All these suggest that CD163 and CD206 identified distinct TAMs. This TAM heterogeneity could stem from their plasticity in response to signals from their microenvironment [28]. A co-culture of CD206-positive TAMs isolated from tumor stroma with tumor cells resulted in the loss of CD206-positive TAM populations [29]. On the contrary, factors secreted by the high-grade tumor cells have been shown to skew TAM differentiation into CD163 TAMs [30]. TAMs occupy distinct spatial niches within the tumor that influence their functions [31,32]. As scavenger receptors, CD163 TAMs function in clear apoptotic debris and engage in endocytosis [33]. They appeared to be more enriched in cases with necrosis.

In terms of prognostication, similar to the results from the other report on digital TAM analysis [16], TAM density alone did not seem to be informative. In addition to TAM density, we assessed also spatial localization. A significantly worse survival rate was observed in patients with low-sCD163 but high-tCD163 TAMs, but not for CD206 TAMs and patients with high sCD163 in both stromal and tumor regions. The intra-epithelial localization of TAMs in other cancers was also correlated with a poor survival [34,35]. Nonetheless, a high level of intra-epithelial TAMs may not always be associated with a poor outcome. Cases with both high tCD163 and sCD163 in our cohort did not have a worse outcome. Of note, these cases showed a higher level of sTIL. sTIL may counteract the adverse impact of the CD163 TAM. In fact, the prognostic value of the CD163 TAM can be refined by their relative proportion to sTILs, and it was shown to be an independent prognostic feature in the current study. The prognostic association with TILs agreed with the immunosuppressive role(s) of CD163 [36]. Consistently, their associations with high environmental gene signatures of suppressive cytokines were reported [20]. Their clinical impact in a neoadjuvant setting has been shown to depend on their spatial association with CD8 [37]. Depending on the environmental stimuli, TAMs could exhibit functional and phenotypic diversity [38]. Therefore, their anatomical localization and interactions with other immune cell types in the TME need to be taken into consideration in their outcome associations.

We did not find the prognostic impact of CD206 TAMs, in contrast to previous studies [13,15]. The precise composition of the immune component in the TME may affect its prognostic impact. Also, there could be a diversity in CD206 TAMs. Notably, emerging data have refuted the assumption that CD206 TAMs are strictly tumor-promoting. Specific macrophage subsets co-expressing CD206 and SERPINH1 or MORC4 were connected with positive patient prognosis in breast cancer [8]. In pre-clinical studies, CD206 TAMs were found to be the primary source of CXCL9—the well-established chemoattractant for CXCR3-expessing NK and CD8 T cells, driving anti-tumor immunity [39]. CD206 TAMs were also shown to have effective antigen cross-presentation capabilities, leading to tumor antigen-specific CD8 T-cell activation [40]. All these results question the validity of CD206 as a marker for protumoral TAMs. TAM-targeting strategies are being tested in cancer therapy [41]. The dissection of TAM diversity and their relative roles in tumors may provide information on strategies to selectively target the protumoral subset. Our study compared the clinical relevance of these two TAM markers, which may provide a rationale for selective macrophage-subset targeting in patients with breast cancer. Caution should be taken in the development of CD206 TAM targeting [42].

Limitations of our study included the small number of cases in subgroups of breast cancer, making it difficult to draw conclusive results from the subtype analysis. sTIL was evaluated based on standard guidelines using H&E slides. The specific subtypes of sTIL in the TME have not been considered. It is not clear if the differences in the sTIL composition could affect the prognostic value of the sCD163/sTIL ratio. The generalizability of the findings can be improved with the evaluation of an independent validation cohort, which was absent in the current analysis. Instead of analyzing the entire section, only selected ROIs in the same areas were used. Although both TAMs shared similar hotspot distribution, there could still be some minor discrepancies in the minority of cases. Moreover, ROI selection could introduce bias to the results.

## 5. Conclusions

In summary, this study revealed the heterogeneity among TAMs in breast cancers, as identified by CD163 and CD206. The two subgroups of TAMs showed distinct clinico-pathologic and prognostic features. CD163 TAMs were more prevalent in high-grade tumors and intra-tumoral locations compared to CD206 TAMs. Furthermore, we have also underscored the significance of the spatial distribution of CD163 TAMs and their interactions with TILs in breast cancer prognosis. In particular, the CD163 TAM-to-sTIL score was determined to be an independent feature in breast cancer survival. Conversely, CD206 TAMs may not have an unfavorable prognostic impact. Further analysis with a larger cohort will be required to validate the current findings in overall breast cancer as well as breast cancer subsets. More investigations, such as those using multiplex imaging, are necessary to better characterize the different TIL populations in relation to the two TAMs, to better define the spatial expression of the two TAMs’ populations and understand the underlying mechanism modulating the TME in breast cancer.

## Figures and Tables

**Table 1:** Clinico-pathological and biomarker correlations of CD163 and CD206 TAM density at different regions.

Features		sCD163		tCD163		sCD206		tCD206	
		LO	HI	LO	HI	LO	HI	LO	HI
**Clinico-pathological features**									
Age	Mean	48.3	49.0	48.7	48.6	47.7	49.3	48.7	48.6
	SD	10.1	10.1	10.1	10.1	9.5	10.9	10.1	10.2
	Median	46.5	48.0	47.0	47.0	46.0	47.5	47.0	47.0
	IQR	41–56	41–45	42–54	40–56	41–54	41–57	41–54	41–56
	Range	30–82	29–75	31–82	29–75	31–75	29–82	31–82	29–75
	*p*	0.972		0.584		0.316		0.818	
Size	Mean	1.89	2.27	1.87	2.29	1.90	2.31	1.87	2.29
	SD	1.06	1.31	1.04	1.31	1.03	1.37	1.04	1.32
	Median	1.60	1.90	1.7	1.9	1.7	1.9	1.7	1.9
	IQR	1.2–2.4	1.5–2.5	1.2–2.2	1.5–2.8	1.2–2.3	1.5–2.8	1.3–2.2	1.5–3.1
	Range	0.2–5.6	0.1–7.6	0.1–5.6	0.2–7.6	0.2–5.6	0.1–7.6	0.1–5.6	0.2–7.6
	*p*	**0.008**		**0.008**		**0.016**		**0.003**	
TIL	Low	101	69	100	70	91	72	100	63
	High (>20)	7	41	8	40	10	36	17	29
	*p*	**<0.001**		**<0.001**		**<0.001**		**0.003**	
Fibrotic focus	No	93	93	89	95	84	92	95	81
	Yes	17	17	19	15	17	16	22	11
	*p*	0.954		0.421		0.689		0.178	
Necrosis	No	93	66	85	74	87	70	93	64
	Yes	15	44	23	36	14	38	24	28
	*p*	**<0.001**		0.058		**<0.001**		0.100	
Apocrine	No	99	97	98	98	93	96	104	85
	Yes	9	13	10	12	8	12	13	7
	*p*	0.393		0.686		0.433		0.393	
Grade	1	12	3	12	3	10	4	11	3
	2	67	52	62	57	56	59	63	52
	3	29	55	34	50	35	45	43	37
	*p*	**<0.001**		**0.006**		0.108		0.209	
LVI	No	101	92	100	93	94	89	104	79
	Yes	7	17	8	16	6	19	12	13
	*p*	**0.032**		0.088		**0.010**		0.404	
LN met	No	57	58	56	59	62	52	67	47
	Yes	47	50	49	48	35	54	48	41
	*p*	0.872		0.792		**0.033**		0.490	
Subtype	Lum A	34	15	30	19	30	20	32	18
	Lum B	60	61	64	57	53	61	67	47
	HER2-OE	4	17	4	17	5	12	8	9
	TNBC	9	16	9	16	13	13	9	17
	*p*	**0.001**		**0.005**		0.149		0.071	
**Biomarkers**									
Ki67	Lo	42	21	38	25	39	24	39	24
	Hi	64	88	68	84	61	82	76	67
	*p*	**0.001**		**0.038**		**0.011**		0.244	
HER2	Neg	102	84	101	85	94	88	105	77
	Pos	4	26	5	25	6	19	10	15
	*p*	**<0.001**		**<0.001**		**0.009**		0.095	
EGFR	Neg	106	105	105	106	98	104	114	88
	Pos	1	4	3	2	2	3	3	2
	*p*	0.369		1.00		1.00		1.00	
ER	Neg	14	37	14	37	19	27	19	27
	Pos	91	72	91	72	81	78	96	63
	*p*	**<0.001**		**<0.001**		0.249		**0.022**	
PR	Neg	17	36	20	33	23	27	20	30
	Pos	88	72	87	73	77	77	95	59
	*p*	**0.004**		**0.036**		0.623		**0.007**	
c-kit	Neg	98	99	97	100	92	98	108	82
	Pos	10	10	11	9	9	9	9	9
	*p*	0.983		0.623		0.898		0.572	
P63	Neg	103	93	99	97	88	101	105	84
	Pos	5	16	9	12	12	7	11	8
	*p*	**0.012**		0.505		0.168		0.845	
CK5/6	Neg	83	89	99	73	88	90	100	103
	Pos	18	18	18	18	20	19	7	7
	*p*	0.849		0.406		0.835		0.957	
CK14	Neg	100	103	102	101	96	99	114	81
	Pos	7	7	6	8	4	9	3	10
	*p*	0.957		0.593		0.197		**0.019**	
PDL1ic	Neg	83	62	84	61	76	65	85	56
	Pos	24	48	23	49	25	43	32	36
	*p*	**0.001**		**<0.001**		**0.020**		0.071	
PDL1T	Neg	87	90	86	91	81	88	96	73
	Pos	9	15	11	13	11	12	11	12
	*p*	0.284		0.800		0.993		0.416	

**Table 2:** Clinico-pathological and biomarker correlations of CD163 and CD206 TAM distance from tumor nest.

Features		sCD163		sCD206	
		LO	HI	LO	HI
**Clinico-pathological features**					
Age	Mean	49.4	47.9	47.5	49.4
	SD	10.1	10.1	10.0	10.3
	Median	48.0	46.0	45.0	48.0
	IQR	42–48	41–54	40–53	43–56
	Range	30–75	29–82	29–75	29–82
	*p*	0.208		0.121	
Size	Mean	2.33	1.81	2.15	2.02
	SD	1.40	0.91	1.34	1.10
	Median	1.90	1.70	1.80	1.80
	IQR	1.4–2.9	1.3–2.2	1.2–2.6	1.4–2.3
	Range	0.2–7.6	0.1–5.6	0.2–7.0	0.1–7.6
	*p*	**0.010**		0.964	
TIL	Low	84	86	84	84
	High (>20)	24	23	23	24
	*p*	0.841		0.897	
Fibrotic focus	No	94	89	93	89
	Yes	14	20	14	19
	*p*	0.275		0.359	
Necrosis	No	79	79	84	74
	Yes	29	30	23	34
	*p*	0.912		0.097	
Apocrine	No	100	95	99	95
	Yes	8	14	8	13
	*p*	0.185		0.260	
Grade	1	5	10	10	4
	2	54	64	57	63
	3	49	35	40	41
	*p*	**0.028**		0.438	
LVI	No	95	98	95	95
	Yes	13	10	11	13
	*p*	0.508		0.700	
LN met	No	61	55	65	52
	Yes	45	50	39	53
	*p*	0.451		0.059	
Subtype	Lum A	24	25	28	22
	Lum B	62	59	61	58
	HER2-OE	10	10	6	12
	TNBC	12	13	10	16
	*p*	0.988		0.247	
Biomarkers					
Ki67	Lo	29	33	32	31
	Hi	78	74	72	77
	*p*	0.547		0.742	
HER2	Neg	90	95	94	92
	Pos	17	13	11	16
	*p*	0.415		0.341	
EGFR	Neg	106	104	105	103
	Pos	1	4	0	5
	*p*	0.369		0.060	
ER	Neg	25	25	18	31
	Pos	82	81	86	76
	*p*	0.970		**0.045**	
PR	Neg	24	27	17	33
	Pos	81	80	87	73
	*p*	0.686		**0.012**	
c-kit	Neg	98	98	98	97
	Pos	9	11	9	10
	*p*	0.670		0.810	
P63	Neg	94	100	100	94
	Pos	14	8	7	13
	*p*	0.177		0.159	
CK5/6	Neg	91	88	94	84
	Pos	16	21	13	23
	*p*	0.400		0.068	
CK14	Neg	100	102	100	100
	Pos	7	7	6	8
	*p*	0.971		0.605	
PDL1ic	Neg	69	75	73	70
	Pos	38	34	34	38
	*p*	0.501		0.596	
PDL1T	Neg	89	87	90	83
	Pos	14	10	9	16
	*p*	0.475		0.134	
sCD163 density	Lo	46	61	51	52
	Hi	61	47	51	55
	*p*	**0.048**		0.839	
tCD163 density	Lo	40	67	45	58
	Hi	67	41	57	49
	*p*	**<0.001**		0.145	
sCD206 density	Lo	49	48	49	50
	Hi	52	53	56	51
	*p*	0.888		0.684	
tCD206 density	Lo	45	68	51	63
	Hi	56	33	54	38
	*p*	**0.001**		**0.045**	

**Table 3:** Multivariate Cox regression on DFS and OS.

Feature *	HR	Lower 95% CI	Upper 95% CI	*p*-Value
OS				
pN	2.866	1.602	5.127	<0.001
sCD163/TIL	3.477	1.238	9.767	0.018
DFS				
Tumor Size	1.588	1.077	2.342	0.020
pN	2.111	1.227	3.632	0.007
sCD163/TIL	1.671	0.983	2.840	0.058

* Features included at the initial step: Age, tumor size, grade, pT, pN, ER, PR, HER2, Ki67 status, TIL status, sCD163-tCD163 subgroups, CD206/TIL (median), sCD163/TIL (median), tCD206/TIL (median), tCD163/TIL (median). Backward stepwise wald method was used and only the last step was shown.

## Data Availability

Data are available upon reasonable request.

## Supplementary Materials

The following supporting information can be downloaded at: https://www.mdpi.com/article/10.3390/cancers16112147/s1. Figure S1. Schematic diagram of digital analysis for CD163 and CD206 staining (A). After IHC staining of CD206 and CD163 (a), the slides were digitalized (b). Annotation of tumor regions and selection of ROI on the scanned image were performed by pathologists (c). Image analysis including cell detection and immunostain quantification was performed using QuPath. Positive cells will be detected based on the optical density of DAB (d). The coordinates of all the detected cells were extracted (e). The spatial and density measurements were further analyzed using QuPath (f). (B) Representative example on ROI selection and cell detection using QuPath. Five ROIs, including two hotspot stromal regions, two hotspot tumor regions, and one representative stromal-tumor interface, were selected and annotated on the digital images by pathologists (a). The image of immunostaining of TAM (DAB; brown chromogen) (b) and the detection results (TAM: yellow, negative: purple) (c) were shown. Inserts showed the magnified view. Figure S2. Representative staining of CD163 and the corresponding staining of CD206 from same area at intra-tumor region (100x). Lower panels showed a high magnification (400x) of the TAM staining in the stromal regions. Figure S3. The density of CD163 and CD206 TAM in different tumor regions. (A) Box-and-whisker plot showing the density of CD163 and CD206 at stromal and tumoral regions (‘x’ indicates the mean value and the line shows the median value). CD163 and CD206 TAM were categorized as high and low subgroups. (B) The 100% stacked bar chart showed the pair-wise comparison between the stromal and tumoral density of CD163 and CD206 TAM according to the low and high subgroups. (C) The stacked bar chart showed the pair-wise comparison between the CD163 and CD206 TAM subgroups at stromal and tumoral regions. Figure S4. The association of clinico-pathological features and biomarkers with TAM density and distance from tumor nest. The 100% stacked bar chart showed the proportion of TAM density/distance from tumor low and high subgroups according to different categories of clinico-pathological features and biomarkers. Chi-square test was used for statistical analysis. Figure S5. The distance of sCD163 and sCD206 TAM from tumor nest. Box-and-whisker plot of the average minimal distance of CD163 TAM and CD206 TAM for each case. (‘x’ indicates the mean value and the line shows the median value). Figure S6. The distance of sCD163 and sCD206 TAM from tumor nest in different breast cancer subtypes. Figure S7. Kaplan-Meier analysis according to TAM densities in stromal and tumor compartments. Figure S8. Kaplan-Meier analysis according to stromal TAM distance from tumor nest. Figure S9. Kaplan-Meier analysis according to grouping based on TAM density in both stromal and tumor compartments. Figure S10. Kaplan-Meier analysis of DFS (A) and OS (B) according to the TAM density in relation to sTIL. Table S1. Condition for IHC staining. Table S2. Parameter setting for QuPath analysis on cell detection and IHC scoring. Table S3. Correlation of CD163 and CD206 TAM.
