# Supplementary material for: Prognostic Significance of CD163+ and/or CD206+ Tumor-Associated Macrophages Is Linked to Their Spatial Distribution and Tumor-Infiltrating Lymphocytes in Breast Cancer
Source: Cancers (Basel). 2024 Jun 5;16(11):2147. doi: 10.3390/cancers16112147 (PMC11172176; doi:10.3390/cancers16112147)

Supplementary Table S1. Condition for IHC staining

Markers	Manufacturer	Clone	Dilution	Antigen Retrieval	Incubation condition (min, °C)	Assessment	Cutoff
ER	Cell Marque	249R-16	1:100	EDTA, microwave	32,37	N	1%
PR	Novocastra	NCL-L-PGR-312	1:200	EDTA, microwave	30,37	N	1%
HER2	Ventana	4B5	Pre-diluted	UCC1, on-board	16,37	M	3+
Ki67	ThermoScientific	RM-9106S	1:200	Citrate, Pressure cooker	32,37	N	20%
CK5/6	Dako	D5/16 B4	1:40	EDTA pH8	32,37	C, M	5%
CK14	Neomarkers	LL002	1:100	EDTA pH8	32,37	C, M	5%
c-kit	Dako	104D2	1:300	EDTA pH8	32,37	C, M	5%
P63	Ventana	4A4	Pre-diluted	EDTA pH8	32,37	N	5%
CD163	Invitrogen	10D6	1:100	CC1	RT, 32	M	-
CD206	Abnova	5C11	1:500	CC1	32,37	M	-

Supplementary Table S2. Parameter setting for QuPath analysis on cell detection and IHC scoring

**Cell detection**
Detection : Hematoxylin OD
RequestedPixelSizeMicrons : 0.5
NucleusBackgroundRadiusMicrons : 8.0
NucleusMedianRadiusMicrons : 0.0
NucleusSigmaMicrons : 1.2
NucleusMinAreaMicrons: 8.0
NucleusMaxAreaMicrons: 300.0
IntensityThreshold: 0.1
maxBackground: 2.0
excludeDAB: false
CellExpansionMicrons: 2.0
includeNuclei: true
smoothBoundaries: true
makeMeasurements: true
**IHC detection**
DAB threshold : >0.48 [Smoothed: 2 µm: Nucleus: DAB OD max]

Supplementary Table S3. Correlation of CD163 and CD206 TAM

			Rs*	p-value	
	tCD163	tCD163		correlation	Paired comparison
SCD163-lo	94	16	0.718	<0.001	0.857
SCD163-hi	15	95			
	tCD206-lo	tCD206-hi			
sCD206-lo	86	17	0.534	<0.001	0.024
sCD206-hi	33	75			
	sCD206-lo	sCD206-hi			
sCD163-lo	76	28	0.493	<0.001	0.579
sCD163-hi	24	77			
	tCD206-lo	tCD206-hi			
tCD163-lo	81	22	0.438	<0.001	0.066
tCD163-hi	36	66			

*Analysis was performed using continuous variables

Supplementary Figure S1. Schematic diagram of digital analysis for CD163 and CD206 staining (A). After IHC staining of CD206 and CD163 (a), the slides were digitalized (b). Annotation of tumor regions and selection of ROI on the scanned image were performed by pathologists (c). Image analysis including cell detection and immunostain quantification was performed using QuPath. Positive cells will be detected based on the optical density of DAB (d). The coordinates of all the detected cells were extracted (e). The spatial and density measurements were further analyzed using QuPath (f). (B) Representative example on ROI selection and cell detection using QuPath. Five ROIs, including two hotspot stromal regions, two hotspot tumor regions, and one representative stromal-tumor interface, were selected and annotated on the digital images by pathologists (a). The image of immunostaining of TAM (DAB; brown chromogen) (b) and the detection results (TAM: yellow, negative: purple) (c) were shown. Inserts showed the magnified view.

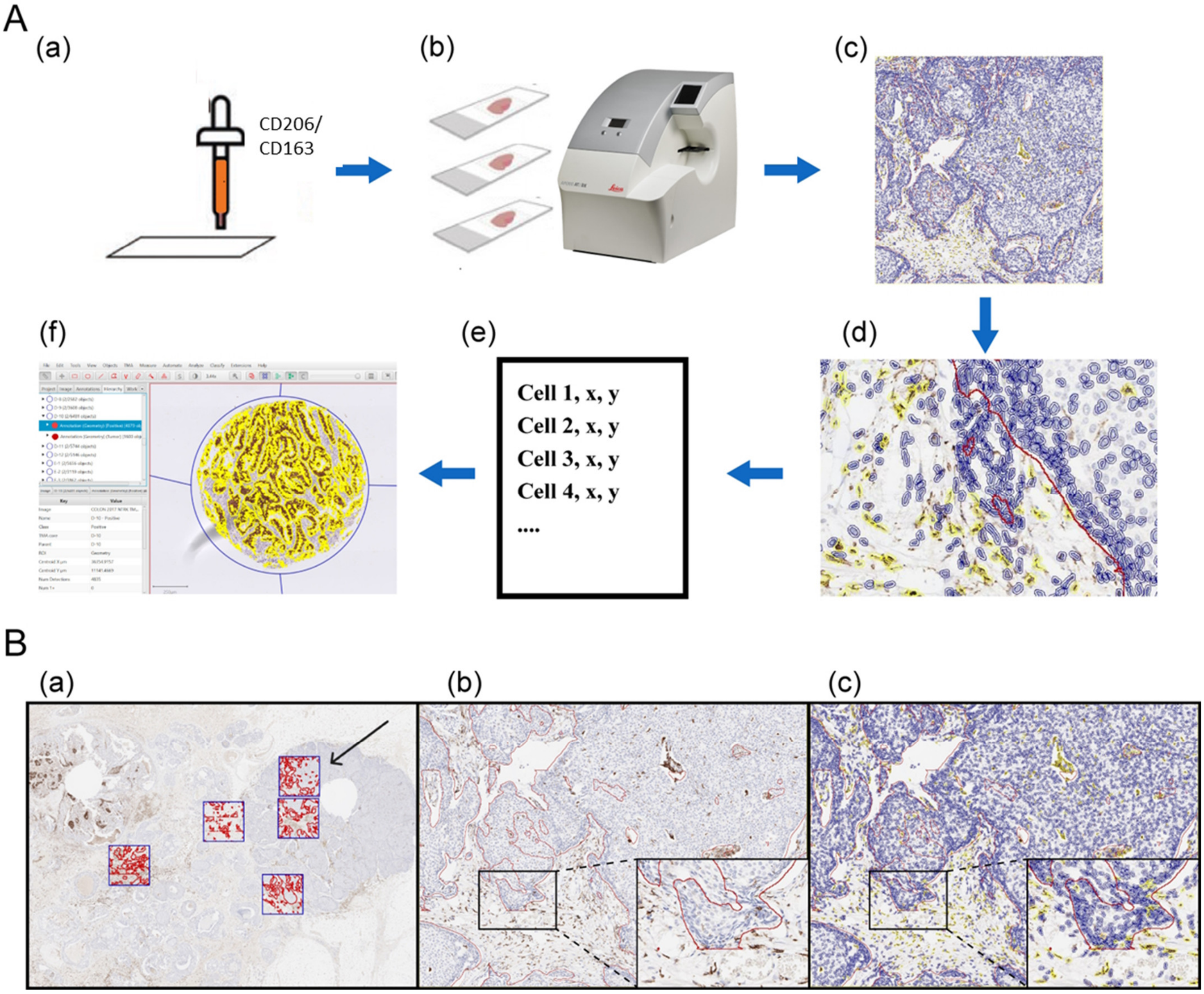

Supplementary Figure S2. Representative staining of CD163 and the corresponding staining of CD206 from same area at intra-tumor region (100x). Lower panels showed a high magnification (400x) of the TAM staining in the stromal regions.

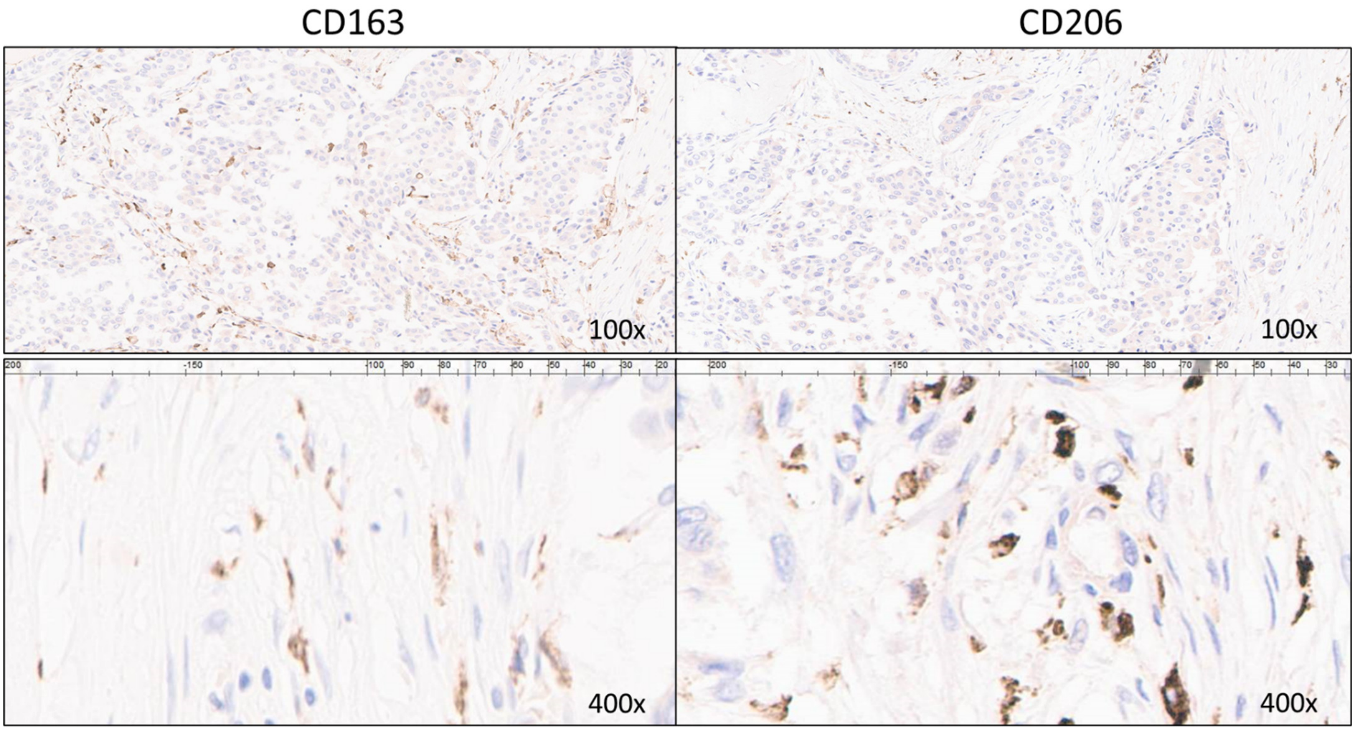

Supplementary Figure S3. The density of CD163 and CD206 TAM in different tumor regions. (A) Box-and-whisker plot showing the density of CD163 and CD206 at stromal and tumoral regions ('x' indicates the mean value and the line shows the median value). CD163 and CD206 TAM were categorized as high and low subgroups. (B) The 100% stacked bar chart showed the pair-wise comparison between the stromal and tumoral density of CD163 and CD206 TAM according to the low and high subgroups. (C) The stacked bar chart showed the pair-wise comparison between the CD163 and CD206 TAM subgroups at stromal and tumoral regions.

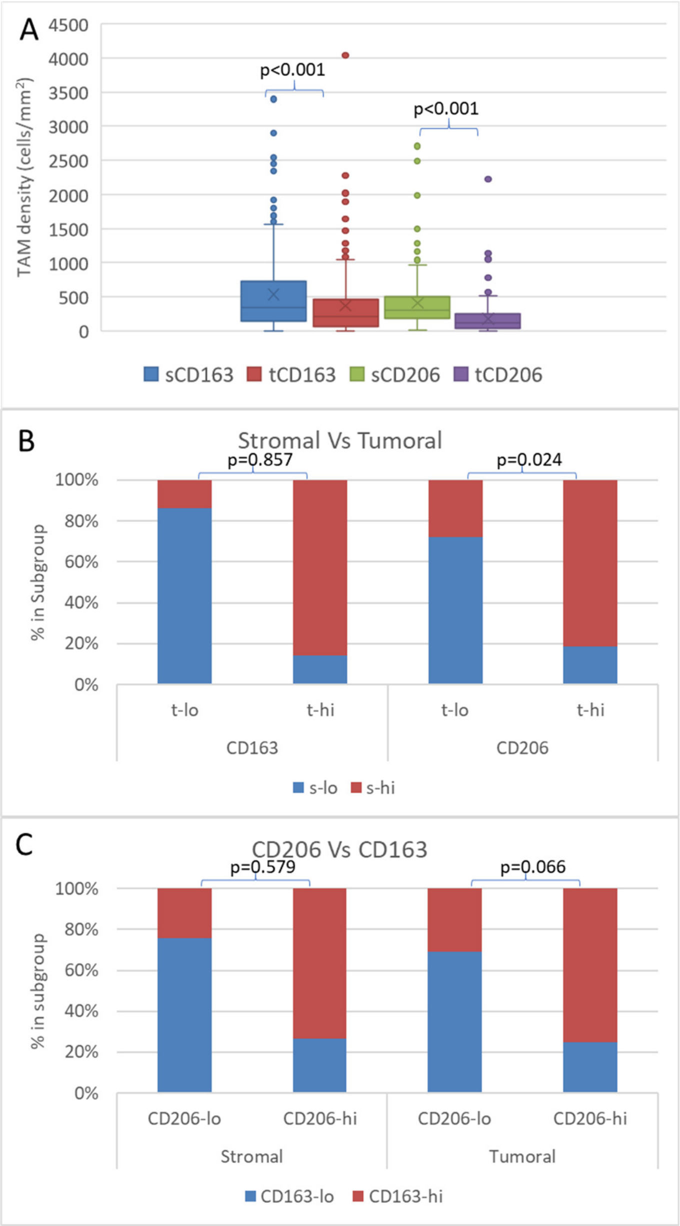

Supplementary Figure S4. The association of clinico-pathological features and biomarkers with TAM density and distance from tumor nest. The 100% stacked bar chart showed the proportion of TAM density/ distance from tumor low and high subgroups according to different categories of clinico-pathological features and biomarkers. Chi-square test was used for statistical analysis. Age/ tumor size

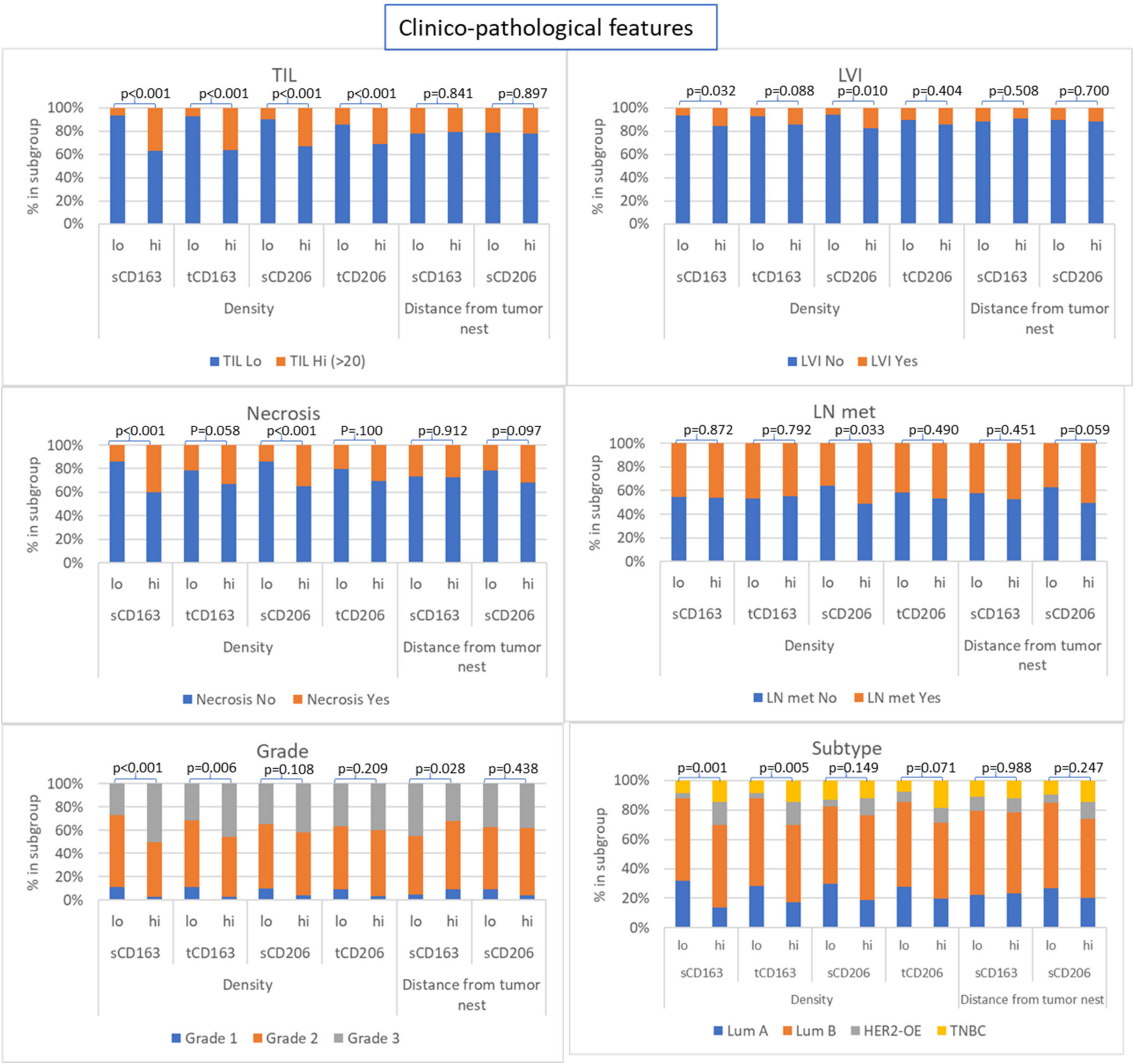

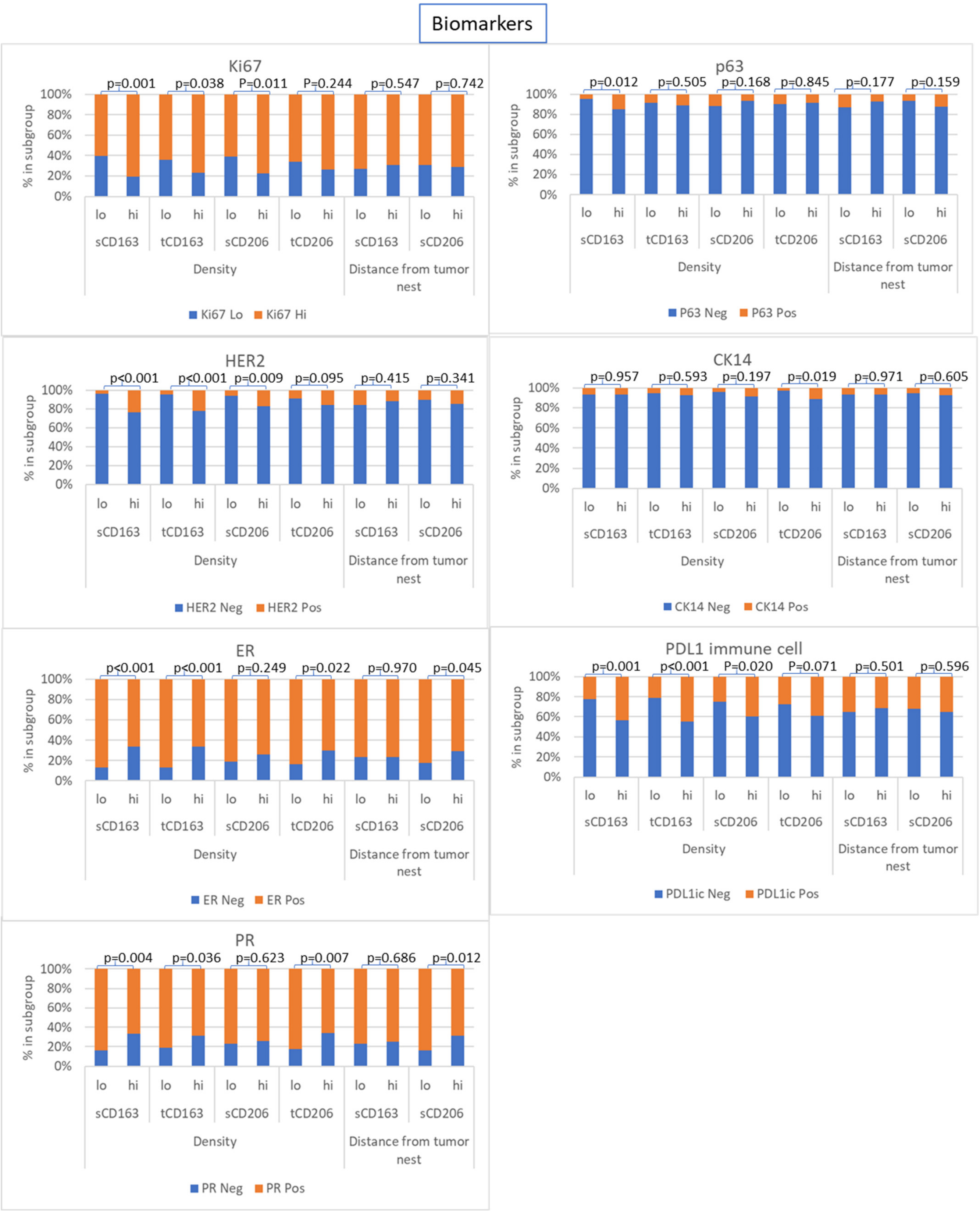
Biomarkers
Ki67
p=0.001 p=0.038 P=0.011 P=0.244 p=0.547 p=0.742
% in subgroup
100% 80% 60% 40% 20% 0%
lo hi lo hi lo hi lo hi lo hi lo hi
sCD163 tCD163 sCD206 tCD206 sCD163 sCD206
Density
Distance from tumor nest
Ki67 Lo Ki67 Hi
p63
p=0.012 p=0.505 p=0.168 p=0.845 p=0.177 p=0.159
P63 Neg P63 Pos
HER2
p<0.001 p<0.001 p=0.009 p=0.095 p=0.415 p=0.341
HER2 Neg HER2 Pos
CK14
p=0.957 p=0.593 p=0.197 p=0.019 p=0.971 p=0.605
CK14 Neg CK14 Pos
ER
p<0.001 p<0.001 p=0.249 p=0.022 p=0.970 p=0.045
ER Neg ER Pos
PDL1 immune cell
p=0.001 p<0.001 P=0.020 P=0.071 p=0.501 p=0.596
PDL1ic Neg PDL1ic Pos
PR
p=0.004 p=0.036 p=0.623 p=0.007 p=0.686 p=0.012
PR Neg PR Pos

Supplementary Figure S5. The distance of sCD163 and sCD206 TAM from tumor nest. Box-and-whisker plot of the average minimal distance of CD163 TAM and CD206 TAM for each case. ('x' indicates the mean value and the line shows the median value).

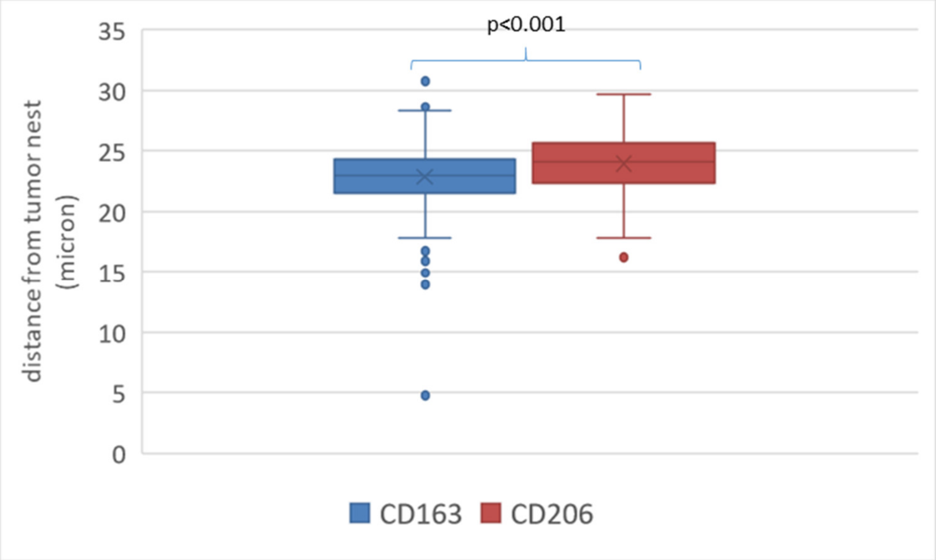

Supplementary Figure S6. The distance of sCD163 and sCD206 TAM from tumor nest in different breast cancer subtypes

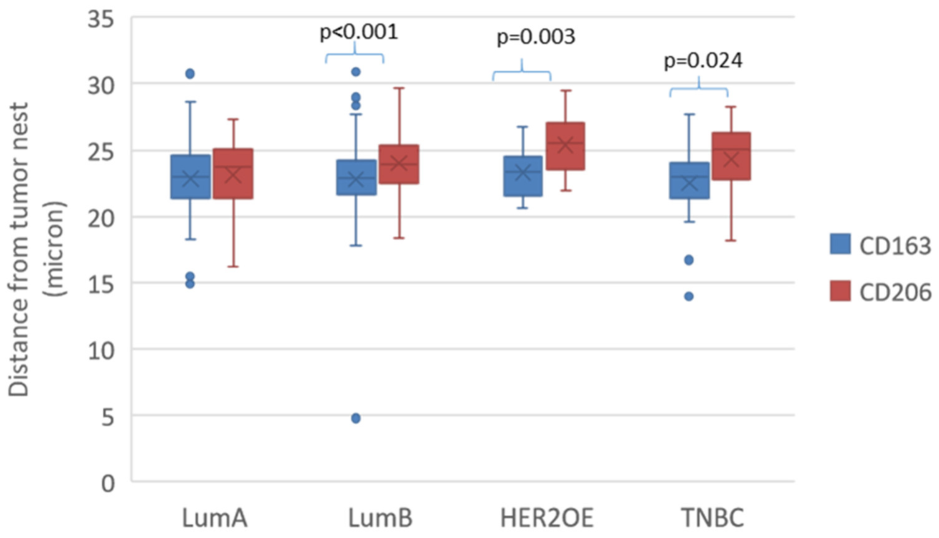

Supplementary Figure S7. Kaplan-meier analysis according to TAM densities in stromal and tumor compartments

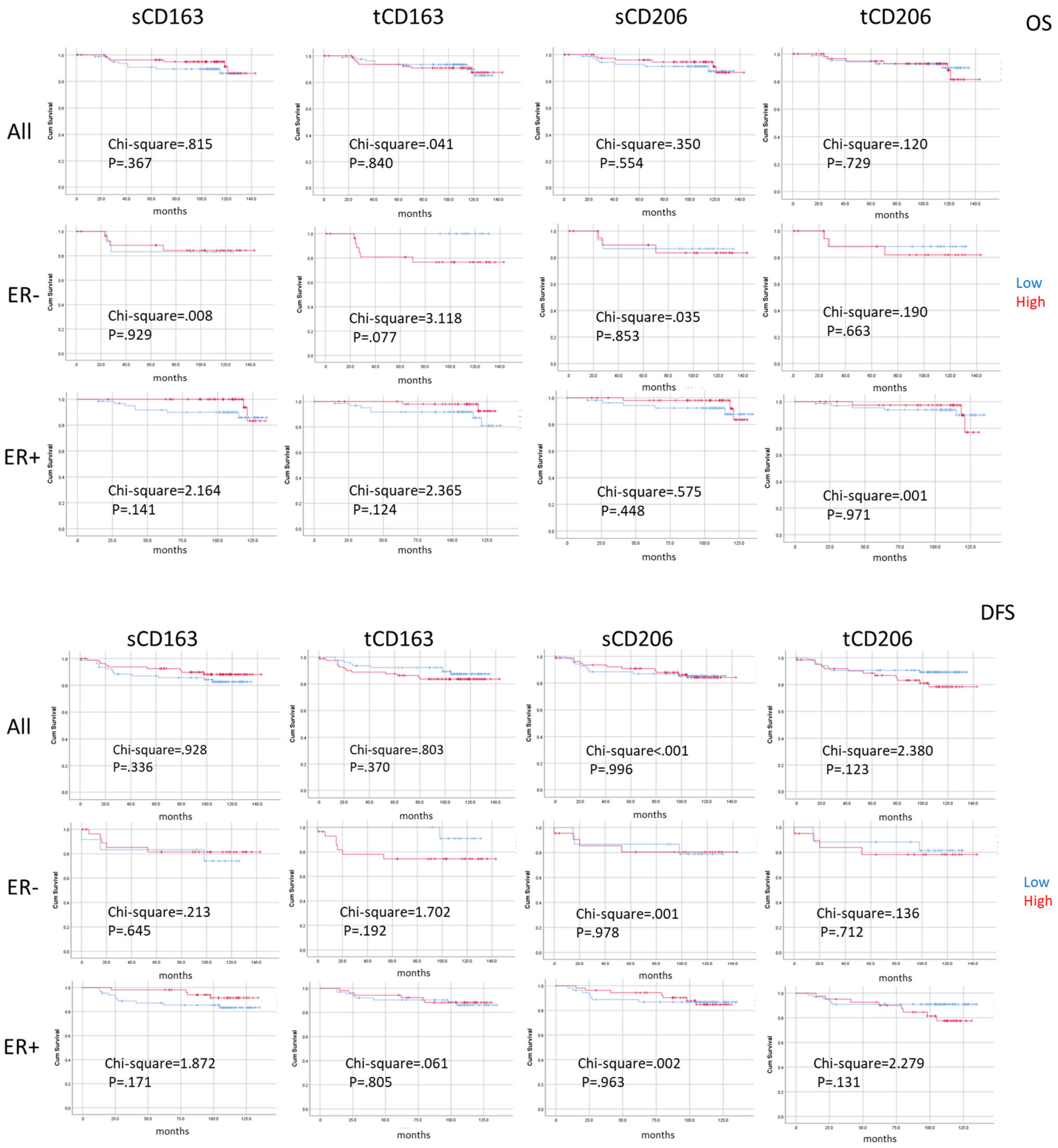

Supplementary Figure S8. Kaplan-meier analysis according to stromal TAM distance from tumor nest

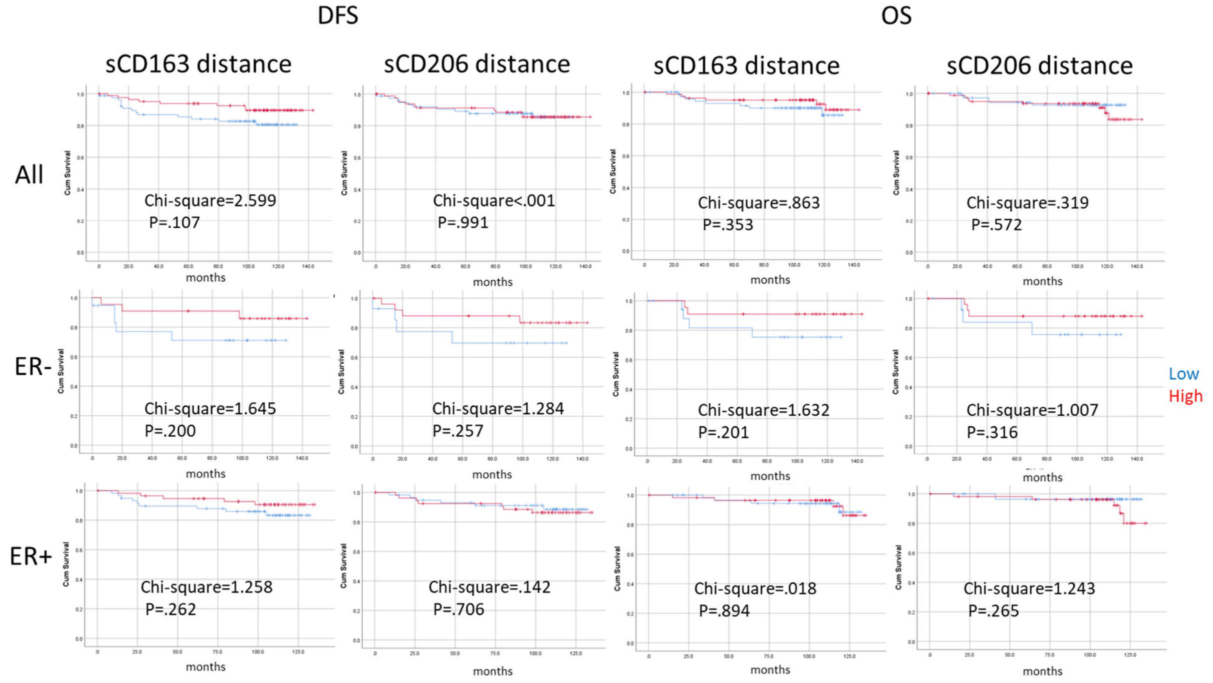

Supplementary Figure S9. Kaplan-meier analysis according to grouping based on TAM density in both stromal and tumor compartments

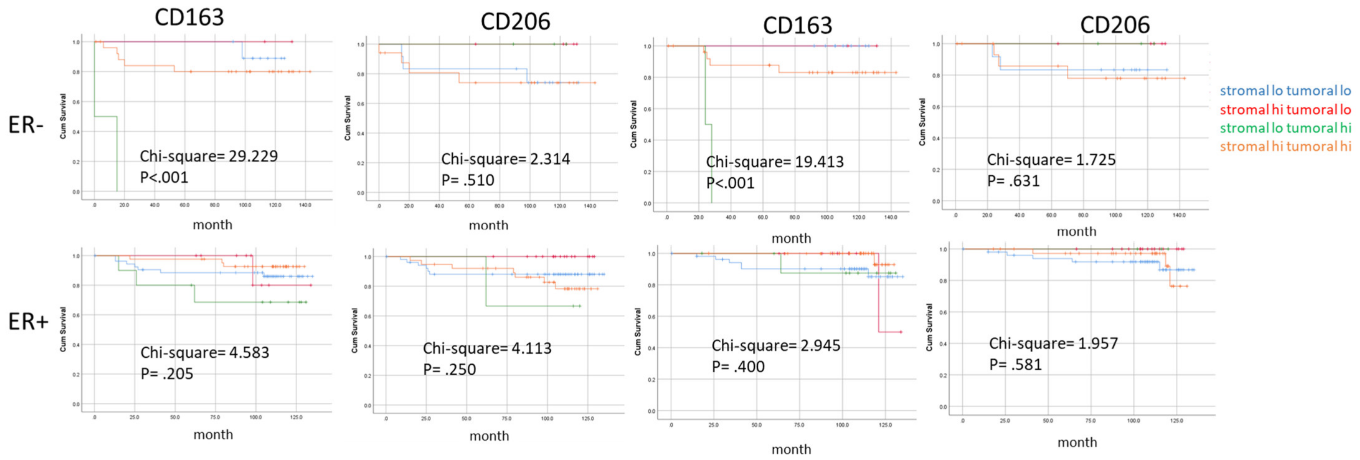

Supplementary Figure S10. Kaplan-meier analysis of DFS (A) and OS (B) according to the TAM density in relation to sTIL

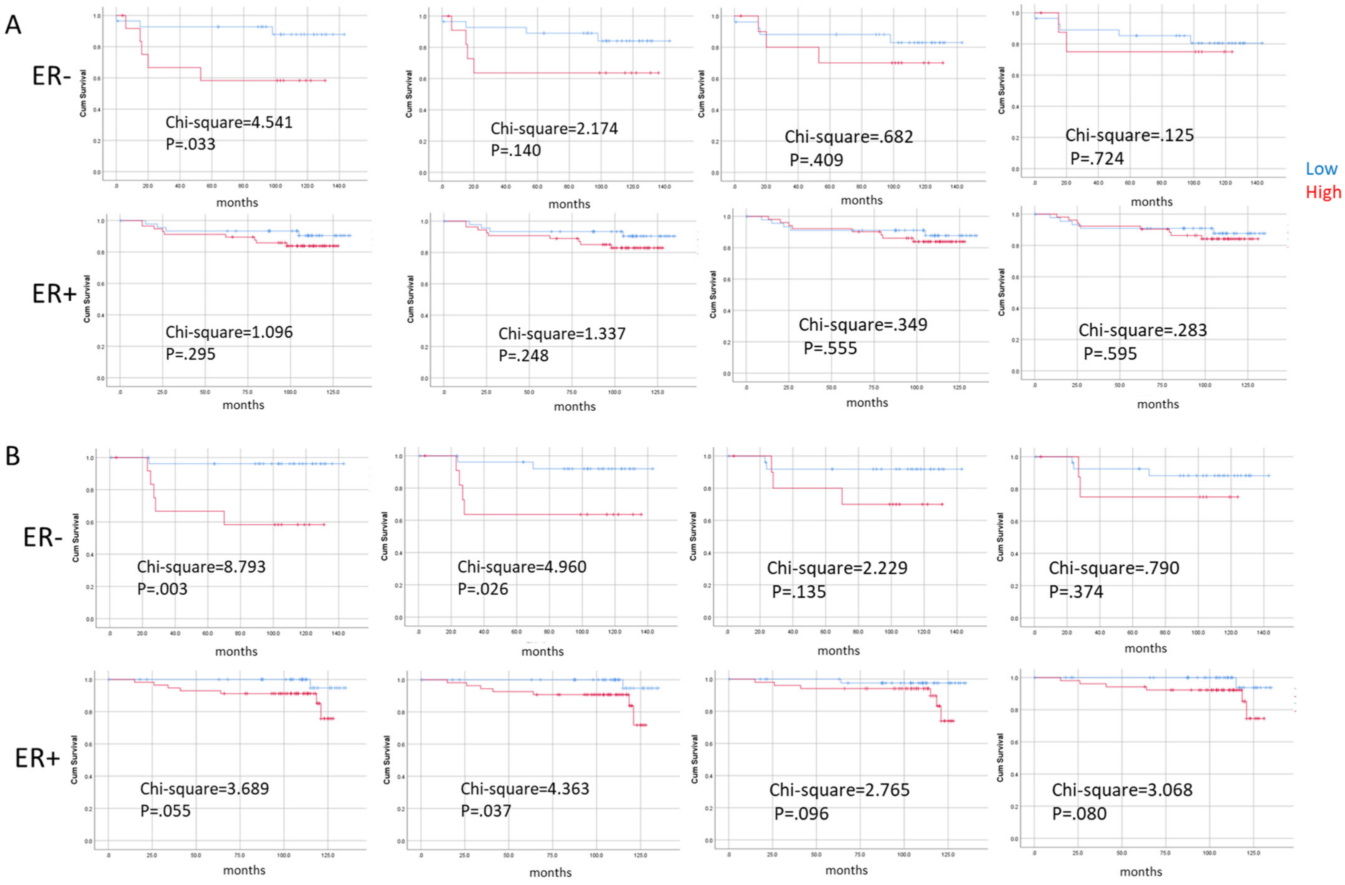

Supplement: Supplementary file 1 [file cancers-16-02147-s001.zip › cancers-3007187-supplementary.pdf]
